# An Enrichment Analysis for Cardiometabolic Traits Suggests Non-Random Assignment of Genes to microRNAs

**DOI:** 10.3390/ijms19113666

**Published:** 2018-11-20

**Authors:** Rima Mustafa, Mohsen Ghanbari, Marina Evangelou, Abbas Dehghan

**Affiliations:** 1 Department of Epidemiology and Biostatistics, Imperial College London, St Mary’s Campus, London W2 1PG, UK; r.mustafa@imperial.ac.uk (R.M.); m.evangelou@imperial.ac.uk (M.E.); 2 Department of Epidemiology, Erasmus University Medical Centre, ′s-Gravendijkwal 230, 3015 CE Rotterdam, The Netherlands; m.ghanbari@erasmusmc.nl; 3 Department of Genetics, School of Medicine, Mashhad University of Medical Sciences, Mashhad 91388-13944, Iran; 4 Department of Mathematics, Imperial College London, South Kensington Campus, London SW7 2AZ, UK; 5 MRC-PHE Centre for Environment and Health, Department of Epidemiology and Biostatistics, Imperial College London, St Mary’s Campus, London W2 1PG, UK

**Keywords:** microRNAs, genome-wide association studies, cardiometabolic, enrichment analysis

## Abstract

MicroRNAs (miRNAs) regulate the expression of the majority of genes. However, it is not known whether they regulate genes in random or are organized according to their function. To this end, we chose cardiometabolic disorders as an example and investigated whether genes associated with cardiometabolic disorders are regulated by a random set of miRNAs or a limited number of them. Single-nucleotide polymorphisms (SNPs) reaching genome-wide level significance were retrieved from most recent genome-wide association studies on cardiometabolic traits, which were cross-referenced with Ensembl to identify related genes and combined with miRNA target prediction databases (TargetScan, miRTarBase, or miRecords) to identify miRNAs that regulate them. We retrieved 520 SNPs, of which 355 were intragenic, corresponding to 304 genes. While we found a higher proportion of genes reported from all GWAS that were predicted targets for miRNAs in comparison to all protein-coding genes (75.1%), the proportion was even higher for cardiometabolic genes (80.6%). Enrichment analysis was performed within each database. We found that cardiometabolic genes were over-represented in target genes for 29 miRNAs (based on TargetScan) and 3 miRNAs (miR-181a, miR-302d and miR-372) (based on miRecords) after Benjamini-Hochberg correction for multiple testing. Our work provides evidence for non-random assignment of genes to miRNAs and supports the idea that miRNAs regulate sets of genes that are functionally related.

## 1. Introduction

MicroRNAs (miRNAs) are small non-coding RNAs of approximately 22 nucleotides that regulate gene expression by either repressing messenger RNA (mRNA) translation or inducing mRNA degradation [1,2]. Over 2000 miRNAs have been identified in humans, which are expected to regulate more than 12,000 genes with over 300,000 validated interactions with target genes [3]. It remains to be understood whether every miRNA regulate genes in random or are organized in a structure related to their affected traits. A recent paper has proposed a so-called “omnigenic model” for the genetic structure of complex disorders, suggesting that several genes act in the core pathways for each trait or disorder and a larger number of genes are connected with these core genes mainly via regulatory networks [4]. The mediators of these networks are suggested to be any structures that regulate the transcriptional process, modify the post-translational phase, or any intercellular signalling processes [4]. In this context, miRNAs might take the mediating role by regulating expression of a group of target genes at the post-transcriptional level [2,4,5].

Enrichment analysis has been shown to be superior compared to single gene analysis in which bias would tend to accumulate across the number of conducted tests [6]. This analysis has been applied in the interpretation of genetic data of several cancers showing that it is superior in revealing common biological pathways of the disease compared to single gene analysis [7]. While this type of analysis has been previously applied by treating the genes as pathways (i.e., grouping the genes associated with one disease as a pathway) [6], we applied enrichment analysis in a different setting by grouping the genes regulated by the same miRNAs as a set of genes. 

Several groups have developed a wide range of gene-enrichment analysis methods to prioritize disease-associated genes in a gene list [7,8,9]. These approaches provide a ranking list of known gene sets as output and scoring the evidence for their associations with a user-defined target gene list of interest [8]. There are also online tools that enable researchers to perform the gene set enrichment analysis [10,11,12,13]. Here we conducted enrichment analysis to investigate the pattern of gene clustering to miRNAs from a disease perspective, particularly whether the grouping is affected by the fact that several genes are associated with the same disease traits. In this study, we chose cardiometabolic traits to study the enrichment of miRNAs for genes related to cardiometabolic disorders.

Cardiometabolic disorders are among complex disorders which share common risk factors, are correlated in their pathophysiology and share underlying pathways [14,15,16,17]. Hundreds of genetic variants identified in genome-wide association studies (GWAS) are increasingly reported for cardiometabolic disorders [18,19,20,21,22,23,24,25,26,27,28,29,30]. Due to the hypothesis-free nature of GWAS, genes that were previously not known to play roles in the disorders have been identified [18,31]. Collectively, the findings are giving more information on the genetic underpinning of cardiometabolic disorders.

We extracted summary statistics from published GWAS for single-nucleotide polymorphisms (SNPs) and genes associated with cardiometabolic traits, including coronary artery disease and blood pressure, lipid, anthropometric and type 2 diabetes and glycaemic traits. Target genes for miRNAs were extracted from online databases. Enrichment analysis was conducted within each database and the results were presented in parallel. We identified miRNAs that are enriched within the cardiometabolic genes, that is, miRNAs that are predicted to have more target genes amongst cardiometabolic genes than what would be expected by chance. We found that genes associated with cardiometabolic traits are clustered into a number of miRNAs, supporting the idea that miRNAs regulate sets of functionally related genes and, correspondingly, that the clustering of genes on miRNAs is not formed on a random basis.

## 2. Results

### 2.1. Retrieval of Cardiometabolic Genes

**Figure 1:**
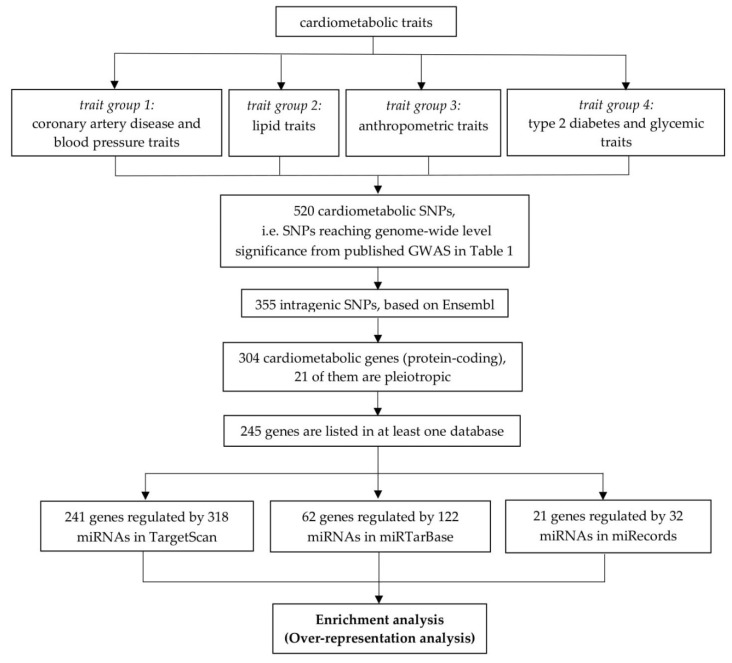
Retrieval of cardiometabolic genes and miRNAs. We extracted 520 SNPs from published GWAS on 20 cardiometabolic traits, of which 355 were intragenic. Those 355 SNPs corresponded to 304 cardiometabolic genes, of which 21 were pleiotropic genes. Enrichment analysis was carried out for cardiometabolic genes and miRNAs within each database (TargetScan, miRTarBase and miRecords) separately.

We retrieved 20,051 protein-coding genes from Ensembl [32] and 610 SNPs reaching genome-wide level significance (*p*-value < 5 × 10^−8^) from published GWAS on cardiometabolic traits (Table 1), resulting in 520 unique SNPs (cardiometabolic-associated SNPs). Two SNPs reported from GWAS (rs9411489 and rs12016871) were not in the Ensembl list as they had been merged with other reference sequence (rs) numbers corresponding to the same location on the genome, so those SNPs were allocated into new rs numbers based on NCBI database (rs635634 and rs9581854, respectively). Twenty-two SNPs were found to occur within the region corresponding to more than one protein-coding genes (Table S1). A total of 355 intragenic SNPs residing in 304 protein-coding genes (cardiometabolic genes) were retrieved. An overview of our approach to retrieve cardiometabolic genes and miRNAs that might regulate those genes is illustrated in Figure 1. 

### 2.2. miRNA Target Prediction Databases

Altogether, we found 245 of 304 cardiometabolic genes to be predicted targets for miRNAs. This resulted in a proportion of 80.6%, which was found to be statistically significant, given on average there are 60.8% of all protein-coding genes that are predicted targets for miRNAs with a standard deviation of 2.78%. We also found a significantly higher proportion of genes targeted by miRNAs (75.1%) for all genes associated with any phenotype as reported from GWAS (Supplementary Method 1, Figure S1, Table S2).

Additionally, we extracted data from Ensembl to calculate the lengths of genes and their three prime untranslated region (3′UTR) of cardiometabolic genes. The steps taken to conduct the analysis and the results are presented in Supplementary Method 2 and Table S3, respectively. We found that, on average, predicted target genes for miRNAs were longer than all protein coding genes. Furthermore, we found that the 3’UTR length was longer in miRNA predicted target genes (2294.1 ± 2240.01 basepair (bp)) in comparison to all genes (1801.0 ± 2063.8 bp). Within the predicted target genes, cardiometabolic genes were found to have a higher average for both gene and 3’UTR length in comparison to non-cardiometabolic genes.

**Figure 2:**
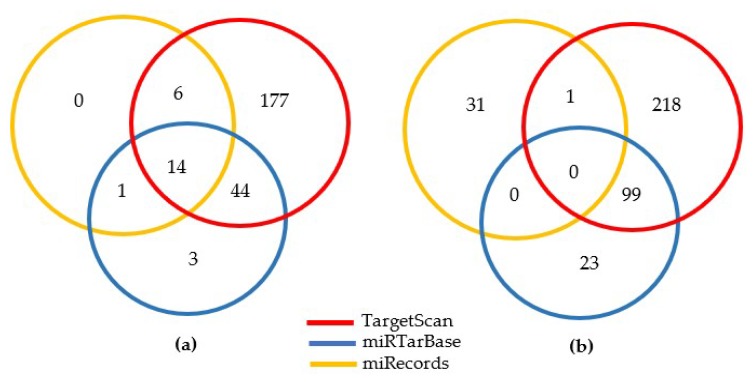
The distribution of cardiometabolic genes (**a**) and cardiometabolic disease-associated miRNAs (**b**) by database (access date: 1 June 2017). (**a**) We extracted 304 cardiometabolic genes from published GWAS, of which 245 are predicted targets for miRNAs; (**b**) A total of 372 miRNAs regulating those cardiometabolic genes were retrieved from the three commonly used miRNA target prediction databases.

A total of 372 miRNAs were found to regulate at least one cardiometabolic genes. We refer to those miRNAs as cardiometabolic disease-associated miRNAs. TargetScan has the widest coverage for cardiometabolic genes and cardiometabolic disease-associated miRNAs, with an average of 607.5 predicted target genes for each miRNA (with a standard deviation of 439.2), in contrast with other databases, with an average of 10.8 and 5.8 in miRTarBase and miRecords, respectively. We identified 318 of 372 (85.5%) cardiometabolic disease-associated miRNAs that are listed in TargetScan, in contrast to only 31 and 122 of 372 (8.3% and 32.8%) cardiometabolic disease-associated miRNAs in miRTarBase and miRecords, respectively (Figure 2). While there is no overlap of cardiometabolic disease-associated miRNAs between those reported in two miRNA validated target databases (miRTarBase and miRecords), we found 99 cardiometabolic disease-associated miRNAs overlap between TargetScan and miRTarBase.

### 2.3. Pleiotropy

**Figure 3:**
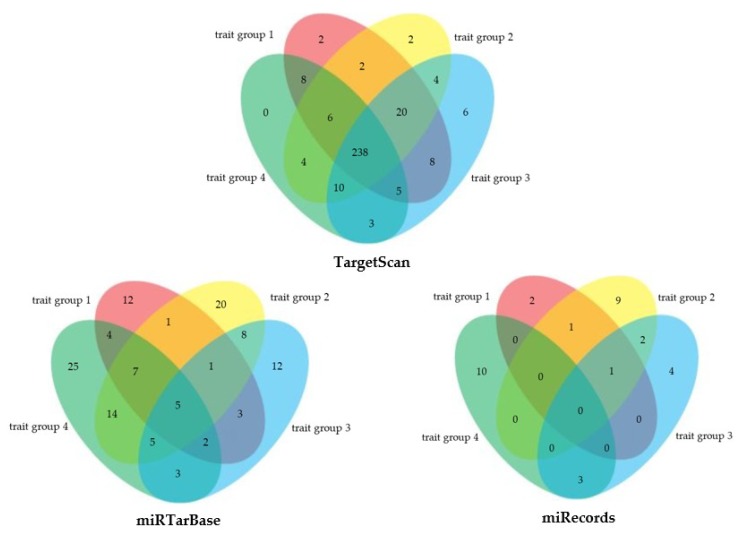
The distribution of cardiometabolic disease-associated miRNAs by trait group. Approximately 74.8% of cardiometabolic miRNAs in TargetScan were predicted to regulate genes that, altogether, associated with four cardiometabolic trait groups. Five pleiotropic miRNAs in miRTarBase were among 238 pleiotropic miRNAs in TargetScan. Trait group 1, coronary artery disease and blood pressure traits; trait group 2, lipid traits; trait group 3, anthropometric traits; trait group 4, type 2 diabetes and glycaemic traits.

Any genes or miRNAs that were found to be associated with more than one cardiometabolic trait groups were defined as pleiotropic. Twenty-one cardiometabolic genes were identified as pleiotropic, consisting of 16 genes associated with two trait groups and five genes (*COBLL1, FAM13A, FTO, HFE* and *SLC39A8*) associated with three trait groups (Figure S2). Of those pleiotropic genes, five genes were reported from both TargetScan and miRTarBase (*ARL15*, *HFE*, *MTMR3*, *SLC39A8* and *TOP1*). Pleiotropic miRNAs were identified for each database (Figure 3), resulting in five pleiotropic miRNAs (miR-125b-5p, miR-155-5p, miR-16-5p, miR-21-5p and miR-23a-3p) associated with all cardiometabolic trait groups in both TargetScan and miRTarBase.

### 2.4. Enrichment Analysis

We conducted enrichment analysis within each database and the results were presented in Table 2. We had conducted preliminary analyses per trait group but the results were not significant due to the small number of genes within each group (Table S4). Our main analysis, therefore, was conducted by considering all cardiometabolic genes within each database. A list of the number of genes in the 2 × 2 table for each database was provided in Table S5. There were 306 miRNAs for TargetScan, 54 miRNAs for miRTarBase and 7 miRNAs for miRecords in the enrichment analysis. Each of those cardiometabolic disease-associated miRNAs was tested within the corresponding database. We identified miRNAs that are predicted to have more target genes amongst cardiometabolic genes than what would be expected by chance, thus are enriched within cardiometabolic genes (Table 2). 

The numbers of miRNAs being analysed in each database were less in comparison to Figure 1 since miRNAs with only one cardiometabolic target gene were not considered in the analysis. When dealing with pleiotropic genes in the enrichment analysis, a total of 21 pleiotropic cardiometabolic genes were found, 20 of which were analysed as they were listed in at least one database (Table S5). When considering all cardiometabolic genes, we identified 102 cardiometabolic disease-associated miRNAs with a significant nominal *p*-value (<0.05) in TargetScan (Table 2). Seven miRNAs appeared to have a significant nominal *p*-value in three databases, while there was no overlap of nominally significant miRNAs between miRecords with other databases (Figure S3). After controlling for false discovery rate (FDR), twenty-nine miRNAs in TargetScan retained significant *p*-values. Altogether, those 29 miRNAs were predicted to regulate 71.8% of cardiometabolic genes. Meanwhile, three miRNAs (miR-181a, miR-302d and miR-372) also retained significant FDR-adjusted *p*-values in miRecords. Altogether, those three miRNAs had been experimentally validated to regulate 19.1% of cardiometabolic genes in miRecords. When dealing with pleiotropic cardiometabolic genes, we observed 19 miRNAs with significant nominal *p*-values in TargetScan, each related to all cardiometabolic trait groups (Table S6).

### 2.5. Biological Interpretation

We found a set of miRNAs that altogether regulate a significantly large proportion of cardiometabolic genes. Previous independent studies have shown these miRNAs as important modulators of cardiometabolic processes (Table 3). Many of the identified miRNAs have been shown in previous experimental and clinical studies to be differentially expressed in cardiovascular and metabolic diseases, suggesting important regulatory roles for these miRNAs on cardiometabolic phenotypes [33,34,35]. At least some of these miRNAs, however, are also associated with pathways that are not specific for cardiometabolic traits [36]. 

miR-181a remained to be significantly enriched in TargetScan and miRecords. Although there were two cardiometabolic target genes for miR-181a in miRecords, multiple cardiometabolic genes were predicted targets for miR-181a-5p, miR-181b-5p, miR-181c-5p and miR-181d-5p in TargetScan. miR-181a-5p was found to have 41 cardiometabolic target genes based on TargetScan. miR-181 family, therefore, may serve as an important miRNA family for cardiometabolic traits supported by the evidence in both predicted and validated databases, hence made it more likely to be a true finding.

## 3. Discussion

In this study, we used existing data from GWAS and miRNA databases to investigate the clustering of genes on miRNAs. We observed that GWAS identified genes and cardiometabolic genes are more likely to be targeted by miRNAs than other protein-coding genes. We further found that the length of the genes and their 3’UTR is significantly higher in GWAS identified genes and cardiometabolic genes which might explain the higher proportion of targeting by miRNAs. However, if the 3’UTR is the main factor contributing to an increased targeting by miRNAs, we would expect that all miRNAs target genes to be slightly enriched for cardiometabolic genes. It is, therefore, hard to conclude if these genes are targeted more by miRNAs due to the longer 3’UTR or whether they had to grow longer during the course of evolution to accommodate regulatory elements such as target sites for miRNAs.

We showed that cardiometabolic genes are clustered as target genes for a limited number of miRNAs, suggesting that the clustering of genes on miRNAs do not occur on a random basis but rather related to their function. Our results agree with the concept of the “omnigenic model” by providing evidence for certain miRNAs as focused regulatory nodes for cardiometabolic genes that might act as mediators in the network of cardiometabolic genes. However, the interaction between genes and miRNAs might not only work one way through gene targeting by miRNAs. Other mechanisms beyond the targeting that affects the interaction might exist, such as the clustering of genetic variants location which has been hypothesized to affect the deregulation of miRNAs [37].

We retrieved cardiometabolic genes reaching genome-wide level significance and the remaining human genes were treated as ‘non-cardiometabolic’. Given the stringent approach in retrieving the cardiometabolic genes from GWAS, we are confident of the findings from our approach. Even though our analysis has provided evidence for miRNAs enrichment for cardiometabolic genes, we found from previous studies that those miRNAs are also associated with other diseases, such as cancers [33]. We expect the same miRNAs might also be enriched for cancer (or any other trait) genes if we had done the analysis for that trait. Our finding, in relation to previous studies, is not surprising given that certain pathways are common among different disorders. Therefore, it is important to look at different pathways in the human body to see if a particular miRNA is a specific regulator for a disease trait [36]. 

Most of our findings in TargetScan could not be replicated in either miRTarBase or miRecords. This could be due to the higher number of predicted target genes in TargetScan, which might have given us a better power to detect enrichments. However, many of the predicted target genes in TargetScan are yet to be validated and reported in miRTarBase or miRecords. Despite their smaller sizes, results from the validated databases could provide additional insight in investigating enrichment for miRNAs. Firstly, because those are based on strong experimental evidence for interaction between genes and miRNAs. Secondly, we conducted the analysis in parallel for three databases so any finding that has been replicated across databases will be more likely to be a true finding. Validated databases curated evidence from experimental studies and provided better coverage on specific miRNAs that are previously known to be associated with diseases. TargetScan, on the contrary, assesses all miRNAs using the same algorithm and therefore is not subject to any bias.

The Context++ scores model in the latest version of TargetScan is suggested to be more predictive compared to other published models [38]. This program predicts biological targets of miRNAs by searching for conserved 7/8-mer sites that match the miRNA seed region. The predictions are ranked based on the putative efficacy of targeting as calculated using Context++ scores of the sites, the higher context score, the greater the probability that a miRNA could target a particular gene [39]. Perfect seed pairing is not a reliable predictor for miRNA-target interactions, which may explain why some putative miRNA target sites were shown to be non-functional. In addition to the bio-informatics analysis of predicting an accessible miRNA-binding site, the functional significance of the predicted miRNA-mRNA interaction needs to be validated experimentally [40]. Although TargetScan takes several features into account for miRNA target prediction, the suggested interactions between miRNAs and genes could be incorrect due to important mechanisms in vivo such as covering the binding sites by folding the mRNA secondary structure or by the molecules bind to the gene 3’UTR. Given that such spurious interactions are not likely to be related with traits and diseases, chances are slim that they have resulted in spurious enrichments in our data. 

A large proportion of GWAS findings reside in intergenic regions, frequently located within or in proximity to regulatory sequences and are thought to have a regulatory function for the genes [41,42]. For many of these findings the genes that mediate the effect are not known. Since those intergenic SNPs are less likely to work through affecting miRNA function, we limited our analysis to SNPs within the protein-coding region of the genes so that the mechanism by which the SNP affects the disease is likely to be through alterations in the host gene expression or functionality. 

We eventually analysed 372 miRNAs with cardiometabolic target genes listed in the available databases, which comprise about one-sixth of all known miRNAs in humans [43]. Any miRNAs that are currently not included in the databases could have affected our analysis and conclusion, hence it is considered as a limitation of our current study. Our analysis is conducted based on the available information from the most recent GWAS of cardiometabolic traits and miRNA target prediction databases. Even though GWAS uses data from hundreds of thousands of individuals, due to the very small effects of each gene, the total percentage of variation that could be explained by the identified genes is not more than a few percents for most traits. This means that there are yet so many other genes to be identified in future GWAS. 

Our analysis has incorporated the biological knowledge within the statistical analysis by grouping the genes regulated by the same miRNAs, rather than treating them individually, thus improving the interpretability of our results, reduce complexity and increase explanatory power [44]. In a similar way, our statistical approach can be applied to analyse larger and more comprehensive datasets, such as the UK Biobank [45], to investigate the genetics of complex disorder.

## 4. Methods

### 4.1. Retrieval of Cardiometabolic Genes

We retrieved SNPs reaching genome-wide level significance (*p*-value < 5 × 10^−8^) in most recent GWAS on 20 cardiometabolic traits conducted by the corresponding consortia (Table 1), which were then referred to as cardiometabolic SNPs. The cardiometabolic traits being included in this study were divided into four groups, namely (1) coronary artery disease and blood pressure traits, (2) lipid traits, (3) anthropometric traits and (4) type 2 diabetes and glycaemic traits. The cardiometabolic SNPs were cross-referenced with Ensembl [32] to identify intragenic SNPs (i.e., SNPs that are located within the protein-coding genes). The protein-coding genes that include the SNPs were then referred to as cardiometabolic genes. Our analysis was restricted to the intragenic SNPs so that the SNPs are likely to play a role in the disease traits by affecting the function of those genes. For SNPs that were not listed in Ensembl, we cross-referenced the information from GWAS with the National Centre for Biotechnology Information (NCBI) database (ncbi.nlm.nih.gov) to identify and use the new rs numbers assigned to those SNPs. All SNPs and genes related information were extracted from Ensembl.

### 4.2. miRNA Target Prediction Databases

TargetScan (v7.1; http://www.targetscan.org/vert_71/), miRTarBase (http://mirtarbase.mbc.nctu.edu.tw/) and miRecords (http://c1.accurascience.com/miRecords/), which are the most widely used databases for miRNA target prediction in previous studies, were used in our analysis [3,39,46]. TargetScan contains the predicted target genes for miRNAs, that is, the genes that are predicted to be regulated by the miRNAs based on computational approach [39]. Meanwhile, for miRTarBase and miRecords, we used the database containing the validated target genes for miRNAs, that is, the genes whose interactions with miRNAs have been supported by experimental evidence [3,46].

Standard miRNA annotation uses a sequential numbering [47]. The term ‘miR’ is applied for mature miRNAs, where for each miRNA precursor there are two mature forms (-3p and -5p) [48]. TargetScan has been following this notation consistently by reporting the mature forms of miRNAs, such as miR-181a-3p and -5p. Some inconsistencies in naming miRNAs, however, were found in the other two databases (miRecords and miRTarBase). We reported any annotation as it is so that further information with regard to any finding in this study can be referred back into the original databases.

The summary of cardiometabolic genes as a result of cross-referencing cardiometabolic SNPs with Ensembl was combined with each miRNA database to summarize the information on the cardiometabolic genes and miRNAs that regulate them, along with their associated cardiometabolic traits. Any miRNAs having cardiometabolic genes as their target were then referred to as cardiometabolic disease-associated miRNAs.

### 4.3. Pleiotropy

We divided twenty cardiometabolic traits into four groups (Table 1). The cardiometabolic genes associated with more than one cardiometabolic trait groups were defined as pleiotropic. Meanwhile, the cardiometabolic disease-associated miRNAs were defined as pleiotropic when they regulate multiple cardiometabolic target genes associated with different cardiometabolic trait groups, thereby resulting in an association between those miRNAs and multiple cardiometabolic trait groups. 

### 4.4. Enrichment Analysis

Since each miRNA regulates a number of target genes, miRNAs were treated as sets of genes in this study. Enrichment analysis was conducted using Fisher’s exact test within each miRNA database separately [49]. We only included miRNAs with at least two cardiometabolic target genes in the enrichment analysis. This restriction was applied to prevent the appearance of miRNAs being significantly enriched due to a false positive finding [50].

As shown in Table 4, a 2 × 2 table was constructed for each of those miRNAs and Fisher’s exact test was carried out for the null hypothesis H_0_(a): the miRNA is not enriched within cardiometabolic genes. For testing H_1_(a) against H_0_(a), we considered protein-coding genes associated with at least one cardiometabolic trait group. The numbers in each cell in the table were calculated independently for each database. For example, in TargetScan, N was obtained by calculating the total number of genes in humans based on TargetScan database. a + b would be the number of all cardiometabolic genes in TargetScan, while a would be the number of cardiometabolic genes in TargetScan that are targeted by the tested miRNA. Additionally, we also conducted enrichment analysis that considered the pleiotropic genes only (protein-coding genes associated with two or more cardiometabolic trait groups), corresponding to the null hypothesis H_0_(b): the miRNA is not enriched within pleiotropic cardiometabolic genes.

In this study, enrichment analysis assessed whether a set of categories (miRNAs) are higher in proportion than what we would expect by chance (i.e., enriched or over-represented) in the subgroup of our data (cardiometabolic genes). Multiple testing issues were corrected with the Benjamini-Hochberg method to control FDR [51]. miRNAs will be considered as enriched when their FDR-adjusted *p*-values are less than 0.05.

### 4.5. miRNA Expression and Association with Cardiometabolic Diseases

We conducted a systematic search using the Human microRNA Disease Database version 3.0 (HMDD v3.0) [33], Human microRNA Expression Database (miRmine) [35] and Human miRNA Tissue Atlas [34] to report any cardiometabolic mechanism/pathways along with any evidence of miRNAs expression on cardiometabolic tissues. We also searched in miRPathDB [36] to find any other pathways linked with the miRNAs, particularly to see if the miRNAs are specific for cardiometabolic traits.

## 5. Conclusions

In this study, we used existing databases and found approximately 80.6% of cardiometabolic genes as targets for miRNAs. We also identified miRNAs that are enriched with cardiometabolic genes. Our finding might indicate a small part of the complex regulatory network of the cardiometabolic genes which supports the idea of non-random assignment of genes to miRNAs and the concept that miRNAs regulate sets of functionally related genes. Similar to pathway analysis, in this work we have added the biological knowledge of miRNAs for improving the interpretability of our results, reduce complexity and increase explanatory power. Our analysis of the currently available data has added new insights that will be integral to the ongoing efforts in studying the roles of miRNAs in complex disorder.

## Figures and Tables

**Table 1:** GWAS related to cardiometabolic traits.

Cardiometabolic Trait	Consortium	Number of SNPs Associated *	Ref
**trait group 1: coronary artery disease & blood pressure traits**			
coronary artery disease	CardiogramplusC4D	51	[19]
systolic blood pressure	ICBP	54	[20]
diastolic blood pressure	ICBP	53	[20]
hypertension	ICBP	11	[20]
**trait group 2: lipid traits**			
total cholesterol	GLGC	39	[21]
triglyceride	GLGC	35	[21]
high-density lipoprotein cholesterol	GLGC	60	[21]
low-density lipoprotein cholesterol	GLGC	30	[21]
**trait group 3: anthropometric traits**			
body mass index	GIANT	108	[22]
waist-hip ratio	GIANT	39	[23]
**trait group 4: type 2 diabetes and glycaemic traits**			
type 2 diabetes	DIAGRAM	18	[24]
insulin sensitivity index (Stumvoll index)	MAGIC	4	[25]
insulin secretion during oral glucose tolerance test	MAGIC	8	[26]
fasting glucose	MAGIC	36	[27]
2 h-glucose	MAGIC	9	[27]
fasting insulin	MAGIC	26	[27]
HbA1c	MAGIC	11	[28]
fasting pro-insulin	MAGIC	9	[29]
HOMA-B	MAGIC	2	[30]
HOMA-IR	MAGIC	7	[30]
**Total**		610	

* The number corresponds to SNPs that reached genome-wide level significance in GWAS. CardiogramplusC4D: Coronary ARtery DIsease Genome-wide Replication and Meta-analysis. (CARDIoGRAM) plus The Coronary Artery Disease (C4D) Genetics Consortium. ICBP: International Consortium for Blood Pressure. GLGC: Global Lipid Genetics Consortium. GIANT: Genetic Investigation of ANthropometric Traits Consortium. DIAGRAM: DIAbetes Genetics Replication and Meta-analysis Consortium. MAGIC: the Meta-Analyses of Glucose and Insulin-related traits Consortium. HOMA-B: homeostasis model assessment for beta cell function. HOMA-IR: homeostasis model assessment for insulin resistance.

**Table 2:** Results of enrichment analysis.

	TargetScan (Predicted)	miRTarBase (Validated)	miRecords (Validated)	miRTarBase & miRecords *
**H_0_(a): the miRNA is not enriched within cardiometabolic genes**				
Number of miRNAs ^#^	306	54	7	61
Nominal *p*-value < 0.05	102	13	3	19
FDR-adjusted *p*-value < 0.05 ^+^	29	0	3	0
**H_0_(b): the miRNA is not enriched within pleiotropic cardiometabolic genes**				
Number of miRNAs ^#^	129	- ^$^	- ^$^	- ^$^
Nominal *p*-value < 0.05	19	-	-	-
FDR-adjusted *p*-value < 0.05 ^+^	0	-	-	-

**^#^** The numbers correspond to miRNAs with at least two cardiometabolic (H_0_(a)) or at least two pleiotropic cardiometabolic (H_0_(b)) genes. ^+^ FDR: false discovery rate. * The analysis included miRNAs whose interactions with their target genes have been validated in either miRTarBase, miRecords or both. **^$^** The analysis could not be carried out since no miRNAs in the databases regulate at least two pleiotropic cardiometabolic genes.

**Table 3:** Biological mechanisms associated with enriched miRNAs.

	Cardiometabolic-Associated Diseases	Cardiometabolic Tissues with Evidence of miRNA Expression
**TargetScan**		
miR-1251-5p	-	pancreas
miR-125a-5p	heart failure, obesity, acute coronary syndrome, coronary atherosclerosis	liver, pancreas, adipocyte, myocardium
miR-125b-5p	myocardial infarction, atherosclerosis, cardiomyopathy, coronary artery disease, heart failure	liver, pancreas, adipocyte, myocardium
miR-1271-5p	type 2 diabetes	liver, pancreas, adipocyte, myocardium
miR-141-3p	ischemic heart disease	liver, pancreas, adipocyte
miR-142-3p.2	type 2 diabetes, heart failure, ischemic heart disease, atherosclerosis	liver, pancreas, adipocyte, myocardium
miR-150-5p	type 2 diabetes, myocardial infarction, obesity, hypertension, heart failure, atherosclerosis, cardiomyopathy	liver, pancreas, adipocyte, myocardium
miR-155-5p	type 2 diabetes, myocardial infarction, cardiomyopathy, coronary artery disease, heart failure, hypertension, acute coronary syndrome, atherosclerosis	liver, pancreas, adipocyte, myocardium
miR-181a-5p	myocardial infarction, atherosclerosis, cardiomegaly, heart failure	liver, pancreas, adipocyte, myocardium
miR-181b-5p	atherosclerosis, diabetic cardiomyopathy, obesity, type 2 diabetes cardiovascular disease (unspecific)	liver, pancreas, adipocyte, myocardium
miR-181c-5p	heart failure	liver, pancreas, adipocyte, myocardium
miR-181d-5p	-	liver, pancreas, adipocyte, myocardium
miR-182-5p	atherosclerosis, type 2 diabetes, heart failure	liver, pancreas, adipocyte, myocardium
miR-186-5p	myocardial infarction, type 2 diabetes, heart failure	liver, pancreas, adipocyte, myocardium
miR-188-5p	cardiovascular disease (unspecific)	liver, pancreas, adipocyte, myocardium
miR-200a-3p	ischemic cardiomyopathy, type 2 diabetes, obesity	liver, pancreas, adipocyte
miR-200b-3p	type 2 diabetes, obesity	liver, pancreas, adipocyte, myocardium
miR-200c-3p	cardiovascular disease (unspecific), type 2 diabetes	liver, pancreas, adipocyte
miR-204-5p	type 2 diabetes, hypertension, myocardial infarction, cardiomyopathy, obesity, heart failure	liver, pancreas, adipocyte, myocardium
miR-211-5p	heart failure	liver, pancreas, adipocyte, myocardium
miR-23a-3p	type 2 diabetes, cardiovascular disease (unspecific), cardiomyopathy, coronary artery disease, heart failure, myocardial infarction	liver, pancreas, adipocyte, myocardium
miR-23b-3p	atherosclerosis, type 2 diabetes, heart failure	liver, pancreas, adipocyte, myocardium
miR-23c	-	liver, pancreas, adipocyte, myocardium
miR-342-3p	heart failure, atherosclerosis, obesity, type 2 diabetes,	liver, pancreas, adipocyte, myocardium
miR-371a-5p	cardiomyopathy	liver, pancreas, adipocyte, myocardium
miR-429	type 2 diabetes, heart failure	liver, pancreas, adipocyte, myocardium
miR-485-5p	-	liver, pancreas, adipocyte, myocardium
miR-493-5p	-	liver, pancreas, adipocyte, myocardium
miR-96-5p	type 2 diabetes, cardiomyopathy	liver, pancreas, adipocyte, myocardium
**miRecords**		
miR-181a	myocardial infarction, atherosclerosis, cardiomegaly, heart failure	liver, pancreas, adipocyte, myocardium (miR-181a-3p and miR-181a-5p)
miR-302d	-	-
miR-372	heart failure	liver (miR-372-3p)

**Table 4:** A 2 × 2 table for Fisher’s exact test.

	Target Gene for miRNA	Not a Target Gene for miRNA	Total
number of cardiometabolic genes	a	b	a + b
number of non-cardiometabolic genes	c	d	c + d
	a + c	b + d	N

The table was constructed to test H_1_(a) against H_0_(a). a: the number of cardiometabolic target genes for the miRNA. a + b: the total number of cardiometabolic genes. a + c: the total number of target genes for the miRNA. N: the total number of protein-coding genes in human. All numbers were calculated independently for each database.

## Supplementary Materials

Supplementary Materials can be found at http://www.mdpi.com/1422-0067/19/11/3666/s1.

## Abbreviations

CardiogramplusC4D	Coronary ARtery DIsease Genome-wide Replication and Meta-analysis (CARDIoGRAM) plus The Coronary Artery Disease (C4D) Genetics Consortium
DIAGRAM	DIAbetes Genetics Replication and Meta-analysis Consortium
FDR	false discovery rate
GIANT	Genetic Investigation of ANthropometric Traits Consortium
GLGC	Global Lipid Genetics Consortium
GWAS	genome-wide association studies
HOMA-B	homeostasis model assessment for beta cell function
HOMA-IR	homeostasis model assessment for insulin resistance
ICBP	International Consortium for Blood Pressure
MAGIC	the Meta-Analyses of Glucose and Insulin-related traits Consortium
mRNA	messenger RNA
miRNA	microRNA
NCBI	the National Centre for Biotechnology Information
RNA	ribonucleic acid
SNP	single-nucleotide polymorphism
