# Supplementary material for: An Enrichment Analysis for Cardiometabolic Traits Suggests Non-Random Assignment of Genes to microRNAs
Source: Int J Mol Sci. 2018 Nov 20;19(11):3666. doi: 10.3390/ijms19113666 (PMC6274774; doi:10.3390/ijms19113666)
Supplement: Supplementary file 1 [file ijms-19-03666-s001.pdf]

SUPPLEMENTARY MATERIALS

An Enrichment Analysis for Cardiometabolic Traits Suggests Non-Random Assignment of Genes to microRNAs

Rima Mustafa [1,†], Mohsen Ghanbari [2,3,†], Marina Evangelou [1,4,5,‡] and Abbas Dehghan [1,5,‡,*]

[1] Department of Epidemiology and Biostatistics, Imperial College London, St Mary's Campus, W2 1PG London, UK; r.mustafa@imperial.ac.uk (R.M.); m.evangelou@imperial.ac.uk (M.E.)
[2] Department of Epidemiology, Erasmus University Medical Centre, 's-Gravendijkwal 230, 3015 CE Rotterdam, The Netherlands; m.ghanbari@erasmusmc.nl
[3] Department of Genetics, School of Medicine, Mashhad University of Medical Sciences, 91388-13944 Mashhad, Iran
[4] Department of Mathematics, Imperial College London, South Kensington Campus, SW7 2AZ London, UK
[5] MRC-PHE Centre for Environment and Health, Department of Epidemiology and Biostatistics, Imperial College London, St Mary's Campus, W2 1PG London, UK
* Correspondence: a.dehghan@imperial.ac.uk; Tel.: +44-20-7594-3347
† These authors contributed equally to this work.
‡ These authors jointly supervised this work.

The Supplementary Materials begin with Supplementary Methods that include a section of text describing two methods to conduct additional analysis in the study. Following them are Supplementary Tables and Supplementary Figures that are referenced specifically in the text.

Supplementary Methods

Supplementary Method 1

From the databases, we found 12,187 out of 20,051 protein-coding genes are targets for miRNAs, which results in a ratio of 60.8%. Further, we found 4000 out of 5329 genes reported from GWAS (associated with any disease trait) that are targets for miRNAs, thus results in a ratio of 75.1%. We conducted the following steps to assess whether 75.1% is statistically different than 60.8%:

1. We randomly selected 5329 genes from the 20,051 protein-coding genes
2. We mapped those 5329 genes to the list of genes that are targets for miRNAs (12,187) compiled from TargetScan, miRTarBase and miRecords.
3. We computed the proportion of those 5329 genes that are targets for miRNAs.

We repeated steps 1 to 3 for 10,000 times to create a null distribution (Figure S1). The summary statistics are presented in Table S2.

Supplementary Method 2

To compare the gene and 3'UTR length between cardiometabolic genes and others, we downloaded data from Ensembl Biomart (http://www.ensembl.org/biomart/martview/a6ebeae0fcc5cc1262568d5f7673f3c3) with the following details:

- Database: Ensembl gene 94
- Dataset: Human genes (GRCh38.p12)
- Attributes: Features; Gene name, Gene start and end (bp), 3'UTR start and end (bp)

The length of genes and 3'UTR of protein-coding genes was calculated as follows:

Gene length (bp) = gene end (bp) − gene start (bp)
3'UTR length (bp) = 3'UTR end (bp) − 3'UTR start (bp)

For genes with more than one transcripts, the longest transcript was selected. The results are presented in Table S3.

Supplementary Tables

Table S1. Cardiometabolic SNPs assigned to more than one protein-coding genes.

SNP	Gene(s)
rs1000940	*RABEP1, NUP88, UGT1A8, UGT1A10, UGT1A9, UGT1A7, UGT1A6, UGT1A5, UGT1A4*
rs11563251	*UGT1A3, UGT1A1*
rs11715915	*AMT, AC104452.1*
rs11776767	*PINX1, AC105001.2*
rs12401738	*GIPC2, DJAB4*
rs12566985	*FPGT-TNNI3K, TNNI3K*
rs1516725	*DGKG, ETV5*
rs17024393	*GNAI3, GNAT2*
rs174546	*FADS1, FADS2*
rs174550	*FADS1, FADS2*
rs17608766	*GOSR2, AC005670.2*
rs17638167	*ELAVL3, AC008481.3*
rs17695224	*FPR3, ZNF577*
rs1808579	*NPC1, C18orf8*
rs2000999	*HPR, TXNL4B*
rs2013208	*RBM5, RBM6*
rs2412710	*CAPN3, AC012651.1*
rs2657879	*SPRYD4, GLS2*
rs3217992	*CDKN2B, AL359922.1*
rs3829109	*CARD9, DNLZ*
rs4765127	*ZNF664, AC068790.8, RFLNA*
rs714515	*DNM3, PIGC*

The information was retrieved from Ensembl (access date: 9 June 2017).

Table S2. The summary statistics from the null distribution reported in Figure S1.

	Value
minimum	0.588
first quartile	0.604
median	0.608
mean	0.608
third quartile	0.612
maximum	0.631
standard deviation	0.006

Table S3. Comparison of gene and 3′UTR length between cardiometabolic genes and other genes.

	Gene Length		3′UTR Length	
	N *	mean±SD (bp)	N $	mean±SD (bp)
All protein-coding genes	19,650	67,906.6 ± 132,464.1	19,058	1801.0 ± 2063.8
All GWAS genes @	5626	111,368.7 ± 192,341.3	5250	2156.3 ± 2273.3
GWAS Cardiometabolic genes	297	157,766.1 ± 230,213.2	296	2570.5 ± 2624.3
Non-cardiometabolic genes	19,353	66,527.6 ± 129,921.4	18,762	1788.8 ± 2051.5
All target genes for miRNAs	12,048	85,287.8 ± 152,367.9	11,991	2294.1 ± 2240.1
GWAS genes targeted by miRNAs	3966	131,699.8 ± 210,648.5	3956	2535.6 ± 2386.8
Cardiometabolic target genes	240	166,791.8 ± 232,857.7	240	2929.6 ± 2740.0
Non-cardiometabolic target genes	11,808	83,631.2 ± 149,841.6	11,751	2281.1 ± 2227.0

* The number of genes with complete information on gene start and gene end from Ensembl (accessed 19 October 2018). $ The number of genes with complete information on 3′UTR start and 3′UTR end from Ensembl (accessed 19 October 2018). @ The genes are reported from the GWAS Catalogue (https://www.ebi.ac.uk/gwas/), bp = basepair, UTR = untranslated region, SD = standard deviation, GWAS = genome-wide association studies.

Table S4. Results of enrichment analysis for each trait group.

	TargetScan					miRTarBase					miRecords				
	All Traits	Trait Group 1	Trait Group 2	Trait Group 3	Trait Group 4	All Traits	Trait Group 1	Trait Group 2	Trait Group 3	Trait Group 4	All Traits	Trait Group 1	Trait Group 2	Trait Group 3	Trait Group 4
Number of miRNAs	306	253	250	268	215	54	6	7	6	10	7	0	0	1	0
Nominal p-value < 0.05	102	0	0	0	0	13	0	0	0	0	3	0	0	0	0
FDR-adjusted p-value < 0.05	29	0	0	0	0	0	0	0	0	0	3	0	0	0	0
Number of genes	241	61	86	72	47	62	17	23	17	12	21	4	8	7	2

Trait group 1, coronary artery disease and blood pressure traits; trait group 2, lipid traits; trait group 3, anthropometric traits; trait group 4, type 2 diabetes and glycaemic traits. FDR = false discovery rate.

Table S5. The number of genes in the 2 × 2 table, by database.

Database	Total Number of Cardiometabolic Genes $	Total Number of Protein-Coding Genes in Human+
	**H_0(a): the miRNA is not enriched within cardiometabolic genes**	
TargetScan	241	11875
miRTarBase	62	2141
miRecords	21	937
miRTarBase & miRecords	68	2521
	**H_0(b): the miRNA is not enriched within pleiotropic cardiometabolic genes**	
TargetScan	20 *	11875
miRTarBase	5 *	2141
miRecords	0 *	937
miRTarBase & miRecords	5 *	2521

$ The numbers correspond to (a + b) in Table 2. + The numbers correspond to N in Table 2. * The numbers correspond to pleiotropic genes only.

Table S6. miRNAs with significant nominal *p*-values from the analysis with respect to H_0(b) in TargetScan.

miRNA	a + c*	a$	*p*-Value	FDR-Adjusted *p*-Value	Associated Trait Group(s) #
miR-1224-5p	200	2	0.0440	0.34	1,2,3,4
miR-154-3p	168	3	0.0027	0.15	1,2,3,4
miR-186-5p	919	5	0.0160	0.26	1,2,3,4
miR-188-5p	214	2	0.0496	0.34	1,2,3,4
miR-192-5p	203	3	0.0045	0.15	1,2,3,4
miR-204-5p	761	4	0.0356	0.34	1,2,3,4
miR-211-5p	761	4	0.0356	0.34	1,2,3,4
miR-215-5p	203	3	0.0045	0.15	1,2,3,4
miR-217	429	3	0.0338	0.34	1,2,3,4
miR-219a-5p	436	3	0.0352	0.34	1,2,3,4
miR-31-5p	460	3	0.0403	0.34	1,2,3,4
miR-339-5p	201	2	0.0444	0.34	1,2,3,4
miR-485-5p	425	3	0.0330	0.34	1,2,3,4
miR-487a-3p	168	3	0.0027	0.15	1,2,3,4
miR-493-5p	768	4	0.0367	0.34	1,2,3,4
miR-873-5p.2	491	3	0.0475	0.34	1,2,3,4
miR-874-3p	313	3	0.0148	0.26	1,2,3,4
miR-875-5p	108	2	0.0140	0.26	1,2,3,4
miR-876-5p	307	3	0.0141	0.26	1,2,3,4

The analysis included miRNAs with a>1. * a + c: total number of predicted target genes for each miRNA. $ a: number of pleiotropic cardiometabolic target genes for each miRNA. # the trait groups associated with the genes which are predicted targets for the miRNA.

Supplementary Figures

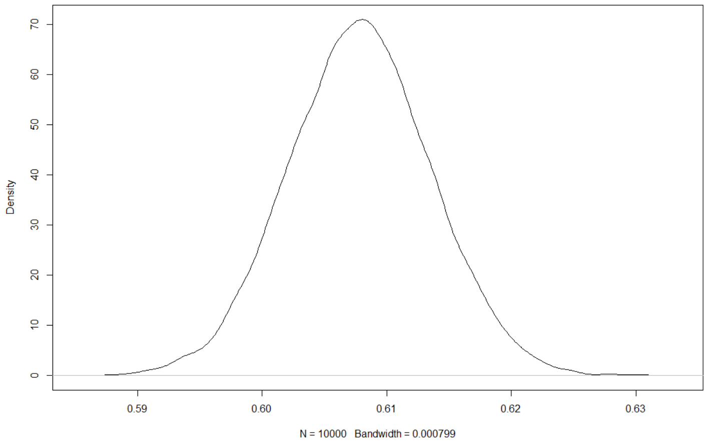

Figure S1. The distribution of the proportion of miRNA target genes.

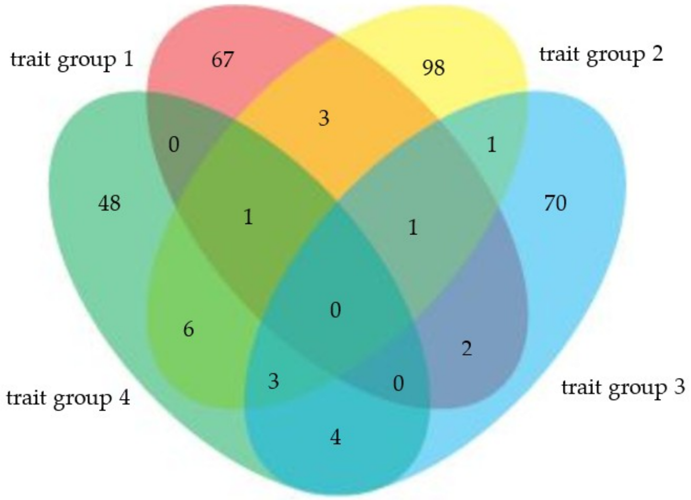

Figure S2. The distribution of cardiometabolic genes by the trait groups (access date: 1 June 2017). Twenty-one cardiometabolic genes were found to be pleiotropic but none of them were associated with all four trait groups.

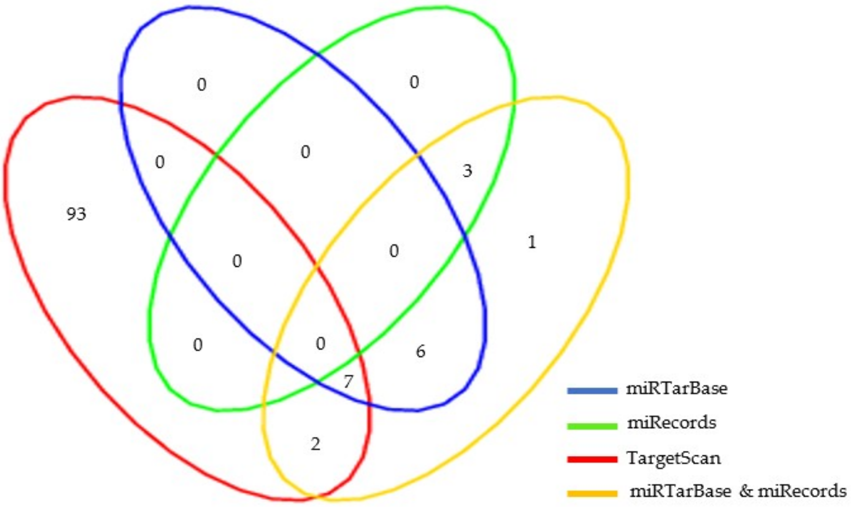

Figure S3. The distribution of cardiometabolic disease-associated miRNAs with nominal *p*-value < 0.05 in the enrichment analysis. Seven miRNAs appeared to have a significant nominal *p*-value in three databases. However, there was no overlap of nominally significant miRNAs between miRecords and other databases.
